# A Decision Which May Cost Dentists Millions

**Published:** 1899-08

**Authors:** 


### Editorial, Etc.

A DECISION WHICH MAY COST DENTISTS MILLIONS. —CROWN AND BRIDGE PATENTS.—COURT HOLDS THAT INTERNATIONAL COMPANY IS ENTITLED TO ROYALTY ON SUCH OPERATIONS.—Some difference of opinion seems to prevail among the dentists of Baltimore in regard to the final results of the decision rendered in New York last Monday by Judge Townsend, of the United States Circuit Court, in favor of the International Crown Tooth Company against James Orr Kyle, a New York dentist. The decision confirms the validity of the patents held by the International Crown Tooth Company, of which Dr. L. T. Sheffield is president, on all operations known as "tooth crowns" and "bridge work." While it is technically against a single individual, it affects almost all of the dentists in the United States.

Litigation extending over 17 years and affecting claims which are estimated at present at $10,000,000 led up to the decision of Judge Townsend. The strongest kind of a fight has been made during the entire period by the complainant company on the one side and by the Dental Protective Association, which includes practically the whole of the dental fraternity of the country, on the other.

### ORIGIN OF THE SYSTEM.

The father of the system of applying tooth crowns was Dr. James Low, a dentist, who performed his first operation in 1877. In 1881 he took out certain patents on the process. At about the same time Dr. Sheffield devoted himself to the problem of the insertion of artificial between sound teeth by what is known as "bridge-work." To perfect the system, Dr. Sheffield purchased from Dr. Low the patents covering his original device, in this way acquiring control over the whole system of operations.

In a short time Dr. Sheffield's establishment became a training school for dentists from different parts of the country who desired to become proficient in the new art, and the system was gradually adopted throughout the country.

At this stage of affairs, to prevent use of the patent without payment of the royalty, it is stated that the patentee sold his right to a syndicate. A stock company was formed, with stock capitalized at $1,000,000. And then the fight began. The company demanded the payment from all who did "crown" and "bridge" work of an annual license of $25 and 15 per cent. of the cost of all operations, fixing also the minimum price for crowning a tooth at $10, but requesting that the usual price be $15.

### BALTIMORE DENTISTS SUED.

Agents of the Sheffield Company were sent out all over the country to prevent any infringement of the patent. During the winter of 1886-87 an agent of the company discovered that Dr. R. B. Winder, who has died since then, and Drs. T. S. Waters, R. B. Roach, A. P. Gore and Marshall Smith, the first dentists in Baltimore to make use of the system of tooth-crowning, performed the operation. They were promptly sued by the company. The suit was not pressed, but the company demanded that the dentists pay the annual license fee and the 15 per cent. on the cost of all operations. They refused to do this, and in a short time joined the Dental Protective Association, which had been organized a short time before by Dr. J. N. Crouse, of Chicago, for the purpose of fighting the company. The Baltimore dentists were not again troubled by the corporation.

Several cases came up and were successfully contested by the Dental Protective Association, the greater part of the dental fraternity throughout the country having joined that association and given it the necessary financial backing.

### QUESTION REOPENED..

Some of the first suits won by the Dental Protective Association were won on the ground that Dr. Sheffield's patents had been anticipated by a Dr. Beardslee. The whole question was reopened in 1898, when the suit was brought against James Orr Kyle. This suit was also defended by the Dental Protective Association, but was lost.

As the patent was taken out in 1881, it expired in 1898. Although the patent expired during the litigation, the decision confirms the right of the complainant company to collect royalties on all infringements covering the whole existence of the patents. Dr. Sheffield claims that tha amount due the tooth crown company on royalties is not less than $10,000,000. Injunction is refused, but the Doctor is given the right to call for an accounting, and it is understood that he will begin at once to collect what he can get out of those whose claims he can establish.

### AN OPPORTUNE TIME.

A Baltimore dentist said yesterday that probably two-thirds of all the dentists in the city now perform the operations of tooth-crowning and bridge-work, and if their claims could be established, it would mean thousands of dollars in the treasury of the Sheffield company. "I attribute the loss of the last case," he said, "to the failure of the dentists throughout the country to support Dr. Crouse.

"However," he continued, "the decision is more opportune at present than it would be at any other time. The National Dental Association is in session now at Niagara Falls. Dr. Crouse is chairman of the executive committee of that body, and if concerted action is to be taken by the dentists throughout the country, the initial steps will probably be taken by the convention."

As an instance of the effective silencing power of the Dental Protective Association, another dentist referred to his experience a little over 10 years ago while practicing in a Western city. He was sued by the tooth crown company for performing the operation of tooth crowning and bridging without paying the prescribed royalty. Not feeling strong enough as a single individual to fight the corporation, he paid the amount of $200, said to be due the corporation, and took out the license. A short time afterward he joined the Dental Protective Association and dropped all connection with the tooth crown company, never removing the first license, but continuing to "crown" and "bridge" teeth. From the date of his enrollment in the protective association all prosecution by the tooth crown company ceased.

### AN APPEAL LIKELY.

Another dentist, who has a large practice in both crown and bridge operations, was inclined to view the matter lightly. He said that he thought without a doubt the case would be carried to a higher court. Until the appeal was made and the decision declared, he said that he refused to despair over it.

"Ethically," he said, "no dentist has any right to patent any contrivance used in his profession. A medical man who would patent a method of a formula would be ignored by all of the best men in the profession. Besides this ethical side of the case, there is the more practical side. The operation of crowning or bridging as carried on to-day is different from the original operation. It has been extended, modified and improved by individual practitioners.

"The trouble with the Dental Protective Association in this last case has been that Dr. Crouse has lost caste by opening a manufacturing association, and some dentists have mistrusted him on that account, and have failed to give the financial support to the protective association that it deserves. Those of us who are acquainted with Dr. Crouse know how time and time again he has neglected his own practice in the interests of the association.

All of the dentists agree that should the present decision be sustained by higher courts it would be extremely difficult to ascertain the indebtedness of any dentist to the corporation. Dentists are not noted for their superior quality of their bookkeeping. Some keep their accounts on slips, which are destroyed on payment of the bills. Some keep their books in cipher, others follow the regular system. Only a few are likely to have the books containing the record for many years back.

In addition to the difficulty in getting at the dentists' records, the dentists claim that to prove the cases the patients would be obliged to go into court to testify that work was done by such a man, in such a place and at such a time, and this they would be unwilling to do.

### METHOD OF COLLECTION.

A prominent lawyer of Baltimore, well acquainted with the procedure in cases of patent infringement, said that the course usually pursued to collect royalties from persons who have used a patented article or method is to send each person a printed circular in which the decision of the courts sustaining the patent right is quoted and asking for an accounting. As a rule, he said, this is sufficient to cause the person who receives the circular to make up an account and settle with the proper persons. This, he added, is likely to be the method pursued by the owners of the patent for "crown" and "bridge" work.

Each dentist in the country will receive a circular and the action of each individual dentist will govern the subsequent proceedings in his case. If he submits a satisfactory statement, together with the sum of money that is due the owner of the patent right, no further proceedings will be taken, but if he refuses to submit a statement, or if his statement is not satisfactory, the owner of the patent right may bring suit against him and require him under oath to make a statement of the amount and value of "crown" and "bridge" work done by him during the life of the patent right.

As a general thing, continued the lawyer, persons who have used a patent right will comply with the demands of its owners and save the heavy court costs.

